# Application of Salivary Biomarkers in the Diagnosis of Fibromyalgia

**DOI:** 10.3390/diagnostics11010063

**Published:** 2021-01-03

**Authors:** Rebeca Illescas-Montes, Víctor J. Costela-Ruiz, Lucía Melguizo-Rodríguez, Elvira De Luna-Bertos, Concepción Ruiz, Javier Ramos-Torrecillas

**Affiliations:** 1 Biomedical Group (BIO277), Department of Nursing, Faculty of Health Sciences, University of Granada, 18016, Granada, Spain; rebecaim@ugr.es (R.I.-M.); vircoss@ugr.es (V.J.C.-R.); luciamr@ugr.es (L.M.-R.); elviradlb@ugr.es (E.D.L.-B.); jrt@ugr.es (J.R.-T.); 2 Instituto Investigación Biosanitaria, ibs.Granada, University of Granada, 18016 Granada, Spain; 3 Institute of Neuroscience, University of Granada, 18016 Granada, Spain

**Keywords:** fibromyalgia, saliva, biomarkers, diagnosis

## Abstract

Fibromyalgia (FM) is a highly prevalent syndrome that impairs the quality of life of the patients; however, its diagnosis is complex and mainly centered on pain symptoms. The study of salivary biomarkers has proven highly useful for the diagnosis and prognosis of numerous diseases. The objective of this review was to gather published data on the utilization of salivary biomarkers to facilitate and complement the diagnosis of FM. Salivary biomarkers used in FM diagnosis include cortisol; calgranulin; and the enzymes α-amylase, transaldolase, and phosphoglycerate mutase. Increased serum levels of C-reactive protein, cytokines interleukin 1-β, interleukin 6, interleukin 8, interleukin 10, interleukin 17, tumor necrosis factor α, and various chemokines may serve as salivary biomarkers, given observations of their increased serum levels in patients with FM. Further research is warranted to study in depth the role and performance of biomarkers currently used in FM diagnosis/prognosis and to identify novel salivary biomarkers for this disease.

## 1. Introduction

Fibromyalgia (FM) is a multifactorial disease that involves the immune-neuroendocrine axis and is typically associated with generalized musculoskeletal pain, pain on digital palpation at “tender points”, lethargy, sleep disorders, anxiety, and/or depression. The severity of symptoms can vary considerably over the course of the disease. FM is not life-threatening and does not have physical sequelae; however, it can severely impair quality of life. It is currently considered a syndrome of unknown etiology, although various causative factors have been proposed, including infection, physical trauma, emotional shock, stress, and even genetics [1,2].

The diagnosis of FM is complex because it has no specific biochemical, imaging, or pathological features. It is usually based on the results of anamnesis and physical examination and obtained by ruling out other diseases. However, involvement of the immune-neuroendocrine axis in this syndrome has prompted study of the diagnostic/prognostic value of different biomarkers [3,4,5]. Information on the biomolecules present in body tissues and fluids contributes to the diagnosis and prognosis of almost all known diseases. Saliva is an especially useful source of clinical data because it can be readily and non-invasively collected [6,7,8]. 

The objective of this study was to explore available data on the usefulness of different salivary biomarkers for the diagnosis and/or prognosis of FM. 

## 2. Fibromyalgia 

FM is the term used to refer to a condition in which the main symptom is generalized chronic musculoskeletal pain that cannot be otherwise explained. It appears to be increasingly prevalent, especially in developed countries; however, it is a complex syndrome associated with multiple symptoms, and its diagnosis is challenging [9,10]. 

The characteristic symptom of generalized pain has been attributed to an anomalous pain perception caused by an alteration of central nervous system and sensory processing and in which the sensitivity of nociceptors changes, with increased excitability and reduced inhibition. This phenomenon has been related to changes in the levels of neurotransmitters, with the inhibition of endorphins, adrenalin, serotonin, endocannabinoids, or glycine and the promotion of glutamate, aspartate, P substance, or neurokinins, among others. Exposure to stress increases the risk of chronic pain, mainly due to changes in the neuroendocrine and autonomic nervous systems [11]. Immune system alterations can also contribute to generalized pain by inducing an inflammatory state [12]. 

The chronic pain in FM patients often varies according to the time of the day, the level of activity, and the weather, and it can be exacerbated by a lack of sleep or high level of stress. FM has been associated with a wide range of heterogeneous symptoms, including tiredness; sleeping disorders; anxiety; depression; poor tolerance to effort; generalized stiffness, especially on awakening; diffuse inflammation on hands and feet; hand tingling; irritable bowel syndrome; abdominal pain/swelling; mouth/eye dryness; urinary disorders; cognitive impairment; headache; and, among women, menstrual pain [13]. Patients with FM frequently report physical or psychological trauma in their past [14], and stress and psychological factors have been implicated in the onset and severity of this disease [11].

Various studies have evidenced the strong association between FM and psychiatric diseases such as anxiety (e.g., panic attacks) or depression [11,14,15,16]. Somatization has been described in a subgroup of FM patients with multiple symptoms unrelated to the disease and with more psychiatric comorbidities [13,17,18]. In this sense, therapies aimed at the management of stress, such as mindfulness-based stress reduction, show an improvement in some of the main symptoms of fibromyalgia and a reduction in the subjective burden of disease [19].

Inflammation is another important clinical symptom of FM. It results from the presence and activity of inflammatory mediators such as proinflammatory cytokines, reactive oxygen species, and plasma-derived inflammatory factors, which may be related to various comorbidities associated with the disease [12,20].

The etiology of FM remains poorly understood, and genetic, environmental (infections, traumas), hormonal, neural, and immunological factors have been implicated, as well as the presence or history of other autoimmune diseases such as arthritis, hypothyroidism, lupus, or Sjögren’s syndrome [1,2,12,20,21,22,23]. The syndrome is designated primary FM when a there is a single disorder and concomitant FM when associated with other diseases [2,24,25]. The co-presence of FM with other clinical conditions has led some authors to claim that FM cannot be diagnosed by ruling out other diseases [26].

FM is much more frequently detected in women than in men (ratio of 12:1), and the highest incidence is observed between the ages of 40 and 60 years [14,27,28]. The prevalence of FM is estimated at 2–4% but varies among countries, regions, and even between urban and rural areas.

Clinical criteria established by the American College of Rheumatology for a diagnosis of FM include a history of unexplained pain for >3 months, combining the generalized pain index (GPI) and the symptomatic severity scale (SS). The GPI measures the number of painful areas from a list of 19, and the SS evaluates fatigue, morning tiredness, cognitive symptoms, and the number of somatic signs [29,30]. However, novel diagnostic strategies are required to complement the current diagnostic procedure, including biomarkers for the differential diagnosis with other conditions that share non-specific symptoms, such as rheumatic polymyalgia, spondylarthritis, inflammatory myopathy, systemic inflammatory arthropathy, and hypothyroidism, among others. The selection of biomarkers should consider four key FM-related factors: pain, emotional stress, oxidative stress, and inflammation. 

## 3. Salivary biomarkers in FM

The main salivary biomarkers used for FM diagnosis/prognosis are cortisol; calgranulin; and the enzymes α-amylase, transaldolase, and phosphoglycerate mutase 1 (PGM1) (Table 1).

### 3.1. Cortisol

Cortisol is a steroidal hormone synthesized from cholesterol, mainly in the suprarenal cortex. Its synthesis is regulated by the hypothalamic–pituitary–adrenal (HPA) axis. In this way, the hypothalamus releases corticotropin-releasing hormone (CRH), which interacts with CRH receptor 1 located in the adenohypophysis, thereby triggering the stimulation and consequent synthesis and release of adrenocorticotropic hormone (ACTH). Finally, the action of ACTH on the fascicular area of the suprarenal cortex augments low-density cholesterol and lipoprotein and increases the activity of cholesterol desmolase, which is responsible for converting cholesterol into pregnenolone, the precursor of steroidal hormones such as cortisol [51,52]. The final cortisol transformation takes place in mitochondrial cytochrome P450, executed by 11ß-hydroxylase (CYP11B1), where 11-deoxycortisol is 11ß-hydroxylated, generating the active form of cortisol [53]. Given that cortisol is a lipid molecule, it binds in the blood to transporting globulin binding proteins (GBPs) and, in a lesser proportion, to albumin; consequently, it circulates in its inactive form, and only 5% of cortisol is present as a free fraction in its bioactive form [54]. 

The best-known function of cortisol in the organism is the regulation of stress when the sympathetic nervous system activates the synthesis of cortisol and other hormones to maintain a state of alertness [55]. It has also been attributed with different actions on the immune response and metabolism regulation [52,56]. Cortisol is also responsible for increasing the availability of blood glucose by gluconeogenesis [57].

Cortisol has been widely analyzed as biomarker in various diseases, with the measurement of free cortisol levels in serum, saliva, hair, or urine [55,58,59,60]. Salivary cortisol is considered valid for research and clinical purposes and has the advantage of being readily sampled in a non-invasive manner [61]. 

As noted above, the etiopathology of FM mainly comprises fatigue, pain, cognitive impairment, and stress [21]. Specifically, a direct relationship has been reported between the primary neuroendocrine response and stress response regulation by cortisol segregation, among others [31]. In this line, Fischer et al. related diurnal fluctuations in salivary cortisol fluctuations to the presence of pain, which was in turn associated with peaks of increased stress in patients diagnosed with FM [33]. Likewise, a direct relationship was found in women with FM between salivary cortisol levels and the presence of depression [36]. It has been observed that the clinical manifestations in the early stages of FM can trigger a greater release of cortisol in comparison to healthy individuals [31,32,62]. For this reason, relaxation and massage therapies have been proposed to reduce pain and stress with the aim of modulating cortisol levels [34,35]. However, some authors detected an anomalous reduction in HPA axis activity at some time after the diagnosis of FM [63], producing hypocortisolism in the patients [37]. Similarly, a meta-analysis of 28 studies comparing saliva, serum, and urine cortisol levels between women with FM and healthy women confirmed a relationship between low cortisol levels and FM due to alterations in HPA axis function [38]. The reduction in salivary cortisol levels was found to be greater with longer duration of the disease [39]. This decrease in cortisol has been described as a possible adaptation to the chronic stress generated by the constant pain endured by the patients [37,40,62].

Various studies have proposed salivary cortisol as FM biomarker; however, it should be taken into account that salivary cortisol levels can be elevated in initial stages of FM, and low level in chronic cases, especially in patients experiencing increased stress.

### 3.2. Enzymes 

Various enzymes have been proposed as diagnostic biomarkers after observations of their changed expression in patients with FM, including α-amylase, transaldolase, and PGM1.

#### 3.2.1. α-Amylase

The biological function of this enzyme of the glycoside hydrolase family is to catalyze the hydrolysis of α-glycosidic bonds of high molecular weight polysaccharides such as starch. It is secreted by various tissues, more abundantly in pancreatic juice and in saliva, being one of the most important salivary enzymes. Besides its function in digestion, salivary α-amylase (sAA) is secreted by acinar cells of the salivary glands in response to stimuli of the autonomous nervous system, specifically the sympathetic nervous system [64]. 

As noted above, stress plays a major role in the physiopathological mechanism of FM. Both the HPA axis and the sympathetic–adrenal–medullary (SAM) axis are physiological mechanisms triggered by stress, and the latter axis also acts by releasing neurohormones [11]. The onset and development of some mental disorders have been related to dysfunctions of these axes [65]. There is increased interest in sAA as an adrenergic biomarker synthesized by stimulation of the SAM axis in response to stress, and various authors have demonstrated a relationship between the stimulation of adrenergic receptors and the secretion of sAA [66,67,68].

Several studies have described significantly increased sAA levels in patients with FM [33,41,42,43]. However, these levels are influenced by the timing of the sampling, which may act as a confounding factor [44]. For instance, Martínez et al. observed higher sAA values in samples gathered from FM patients at midday and night than in those collected in the morning, while values at all three times were above the normal range [42]. This may explain the variability in published results and the finding by some authors of no significant relationship among sAA level, pain, and stress in patients with FM [33]. However, attempts to reduce α-amylase levels in these patients with stress-control strategies using music therapies or physical activities have proven unsuccessful [69,70].

Stress is known to exacerbate pain and vice versa. Ahmadi-Motamayel et al. and Vahedi et al. both found a correlation between pain intensity and sAA level, suggesting that this could be a good biomarker to quantify pain intensity [45,46]. However, Fischer et al. evaluated sAA levels as an indicator of stress-sensitive systems, measuring momentary stress and pain levels and gathering saliva samples at the same time daily for 14 days from 32 women with FM [33]. They found that the relationship between momentary stress and pain was mediated by cortisol but not by α-amylase.

In summary, sAA can be considered as a non-invasive biomarker that can be useful, alongside cortisol, to evaluate the functioning of HPA and SAM axes in response to stress in patients with FM. However, further studies are required to fully elucidate their involvement in symptom modulation and disease development.

#### 3.2.2. Transaldolase

This homodimeric enzyme participates in the pentose phosphate pathway, and its main function is to generate nicotinamide adenine dinucleotide phosphate (NADPH) and five-carbon sugars used to synthesize nucleotides and nucleic acids [71].

In both two-dimensional electrophoresis (2-DE) and Western blot studies, Bazzichi et al. observed transaldolase overexpression in women with FM [47]. This significant increase in expression may be due to increased oxidative stress at tissue level in patients with FM. This can involve glutathione, a protective agent against oxidative stimuli, in whose metabolism NADPH actively participates for its functional recovery [72,73]. Bazzichi et al. constructed receiver operating characteristic curves to assess the diagnostic accuracy of their results and capacity to discriminate between healthy individuals and FM patients, concluding that transaldolase is a useful biomarker of this disease [47]. Thermal treatments (balneotherapy or mud therapy) had no effect on transaldolase expression in women with FM [49], although these therapies act to reduce oxidative stress by increasing the concentration of antioxidants such as coenzyme Q10- CoQ (10-OX) and alpha-tocopherol [74].

Ciregia et al. confirmed the increase in transaldolase expression in a 2-DE study of patients with FM, although this expression did not differentiate between FM and migraine [48]. 

#### 3.2.3. Phosphoglycerate mutase 1

This widely distributed intracellular protein, with a molecular weight of ≈29kDa, participates in the glycolysis metabolic pathway, which generates ATP from the oxidation of a glucose molecule. PGM1 acts in this pathway by catalyzing the interconversion of monophosphoglycerates (3-phosphoglycerate to 2-phosphoglycerate) [75].

Bazzichi et al. used ELISA and Western blot techniques to demonstrate overexpression of PGM1 in patients with FM [47]. This enzyme was studied by Ciregia et al. in saliva from healthy individuals, migraine sufferers, patients with rheumatoid arthritis, and patients diagnosed with FM, employing ELISA and 2-DE techniques [48]. According to the results, PGM1 is a good biomarker of FM but is also increased in patients with migraine. The expression of PGM1 was markedly decreased in patients with FM after 12 weeks of mud-balneotherapy, unlike the expression of transaldolase [49].

### 3.3. Calgranulin

The name of these proteins of the S100 family reflect their capacity to dissolve in a saturated ammonium sulfate solution [76]. Calgranulin A and calgranulin C, also known as S100A8 and S100A12 proteins, respectively, have been proposed as diagnostically useful for FM [77,78]. Both proteins are secreted by neutrophils, monocytes, and macrophages, and their functions include the regulation of inflammatory processes, favoring the secretion of proinflammatory cytokines, reactive oxygen species, and nitric oxide; they can also modulate cell proliferation, differentiation, and apoptosis and maintain calcium homeostasis and cytoskeleton, and they possess antimicrobial properties [77,78,79,80,81].

Bazzichi et al. used the 2-DE technique to reveal overexpression of calgranulin A and C in patients with FM in comparison to healthy individuals [47]. These results were verified in a mass spectrometry study by Giacomelli et al., finding increased levels of both biomarkers in the saliva of patients with FM. This increase was attributed to a possible protective role of these molecules against oxidative stress [50].

### 3.4. Other Biomolecules

The expression of other biomolecules is increased in FM, but their role in the physiopathology of this disease has not been elucidated. This is the case of cyclophilin A, which is involved in the correct folding of other proteins through its peptidylprolyl isomerase enzymatic activity. It is known that cyclophilin A can be secreted by smooth muscle cells and macrophages in response to oxidative stress, which may explain its overexpression in women with FM [82,83]. Other molecules overexpressed in women with FM include Rho GDP-dissociation inhibitor 2, which regulates cell morphology and the motility of non-transformed cells [84]; proteasome subunit-α-type-2, which is related to the proteolysis of most intracellular proteins [85]; and haptoglobin-related protein precursor, related to the innate immune response [86], although its mechanism of action remains unknown.

## 4. Potential Salivary Biomarkers in FM

Various candidate FM biomarkers have been studied in serum or plasma but not in saliva, despite the multiple advantages of this matrix [6,7]. Among these, C-reactive protein (CRP) and different cytokines have demonstrated high diagnostic potential for this disease (Table 2). 

We highlight the role of mast cells in the secretion of some of these cytokines and in the development of mechanisms to increase their levels in FM. Mast cells from the thalamic area secrete interleukin (IL)-6 and tumor necrosis factor (TNF), mediators that activate microglia in the thalamus nucleus or ascending nociceptive pathways, generating the sensation of pain [87].

### 4.1. C-reactive Protein 

This globulin of the pentraxin family [108] is mainly synthesized and secreted by hepatocytes due to an increase in proinflammatory cytokines (e.g., IL-6, IL-1, and TNF-α) in response to acute and chronic inflammatory processes. CRP has demonstrated diagnostic and prognostic value in various diseases [109] and has been found to provide information on the inflammatory status of patients with FM, being proposed as a biomarker of this disease [88,89,90,110]; however, its role in FM is poorly understood.

### 4.2. Cytokines 

Cytokines play a major role in physiological mechanisms in the majority of organs and systems, including the immune system [111]. Imamura et al. described the role of certain cytokines in FM [91], and Wallace et al. associated their action with the onset of FM-related symptoms, especially chronic pain [92]. Some hypotheses on the mechanisms underlying the effect of this disease on the central and peripheral nervous system propose that interleukins and their role in nociception are determinant factors in the development and persistence of FM [87]. The following cytokines have been studied in different fluids in relation to FM: IL-1 β, IL-6, IL-8, IL-10, IL-17, TNF-α [112], and various chemokines.

#### 4.2.1. IL-1 β

This cytokine is considered an important mediator in the inflammatory response. In the central nervous system, macrophages and monocytes in microglia are responsible for IL-1 β production and secretion [113]. It participates in numerous cell mechanisms, including proliferation, apoptosis, and differentiation. Account should be taken of the role of the IL-1 family in the differentiation of Th-17 cells, which are involved in numerous autoimmune and chronic inflammatory diseases [114]. Elevated levels of IL-1 β [91] and IL-1 Receptor antagonist [97] have been reported in serum from patients with FM and increased levels of IL-1 β in skin samples [95], although no relationship between IL-1 β levels and FM was found by other authors [92,93,94,96]. Likewise, Cê et al. did not observe altered IL-1 β salivary levels in patients with fibromyalgia [115].

#### 4.2.2. IL-6

This important proinflammatory cytokine is produced by numerous cell lines and is related to a wide range of physiological processes, autoimmune diseases, and neoplasms [116,117]. IL-6 is involved in the onset and persistence of pain, and elevated levels have been found in patients with chronic pathological pain [118,119]. Many studies have described increased serum/plasma IL-6 levels in FM patients [5,91,96,97,98,99], and these have even been correlated with the intensity of pain [100]. Elevated levels of this biomolecule have also been observed in skin biopsies from FM patients [95] and in their peripheral blood mononuclear cells [92], which was found to be related to hyperalgesia, fatigue, and depression. Geiss et al. described increased IL-6 levels with the stimulation of pressure pain thresholds at tender points in women with FM and associated these levels with the pain and fatigue reported by the patients [101]. However, the mechanism of action of this biomolecule in FM is not well understood.

#### 4.2.3. IL-8

This inflammatory cytokine is produced by a wide range of cell types and is most frequently secreted in response to inflammatory stimuli [120,121]. The presence of this inflammatory biomolecule in the serum of FM patients is well documented [91,92,96,102,103]. In addition, IL-8 levels have been related to reflex sympathetic pain [92] and to the severity and development of pain [5,104]. Kadetoff et al. found elevated IL-8 levels in the serum and cerebrospinal fluid of FM patients [93]. Although Kutu et al. found no significant differences in serum IL-8 levels between FM patients and controls, these levels were related to the severity of pain [94].

#### 4.2.4. IL-10

This anti-inflammatory cytokine inhibits the production of proinflammatory cytokines such as interferon-γ (IFN-γ), TNF-α, IL-1β, and IL-6 in various cell types [122,123]. The detection of elevated IL-10 levels in the serum of FM patients led to its proposal as an FM biomarker [91,102,105,106], although some authors found no relationship between levels of this cytokine and FM [92].

#### 4.2.5. IL-17

This pro-inflammatory cytokine is produced by Th-17 cells and participates in inflammatory processes, promoting the production of chemokines and granulocyte colony-stimulating factors and mobilizing immune cells to infection sites [112,124]. Elevated serum levels of this cytokine have been widely reported in FM patients [99,106], and some authors have related its presence to characteristic symptoms of the disease, such as anxiety [125] and pain [126].

#### 4.2.6. TNF-α

This proinflammatory cytokine has an important function in acute and chronic inflammatory processes [112], and TNF receptor 1 plays a key role in nociceptive signaling [11,127]. Elevated TNF-α levels in sera [96,98,102,105,106] or skin biopsies [95] have been related to the presence of FM, although other authors found no significant differences in serum TNF-α levels between FM patients and controls [91,92,94]. However, Kutu et al. found a positive correlation between TNF-α levels and pain severity [94].

#### 4.2.7. Chemokines 

These cytokines actively intervene in cell migration in response to various stimuli. They are classified into four categories according to their functional capacity and the receptor family with which they interact (CC, CXC, CX3C, or XC). Chemokines participate in pain modulation and regulation via direct mechanisms related to neuron activation or via indirect mechanisms related to leukocyte activation [128]. Chemokines therefore have an important role in peripheral and central sensitization by activating nerve cells of both central and peripheral nervous systems. This phenomenon may help to explain the participation of chemokines in FM pathogenesis, given that higher serum chemokine levels have been observed in patients with FM than in healthy individuals [12,92,107,111,129]. 

## 5. Conclusions

In conclusion, salivary levels of various biomarkers are known to change in the presence of FM and can therefore be useful for its diagnosis as a complement to clinical findings. These biomarkers include cortisol, calgranulin, and the enzymes α-amylase, transaldolase, and PGM1, which have been related to characteristic FM symptoms of stress, pain, and oxidative stress. Other inflammation-related markers are elevated in the serum of patients with FM and are proposed as potential salivary biomarkers of this disease, with the clinical advantages of saliva as sample matrix. Further in-depth study of known biomarkers of FM is needed, and continued research efforts are warranted to identify novel and reliable salivary biomarkers.

## Figures and Tables

**Table 1:** Salivary biomarkers involved in fibromyalgia diagnosis.

Biomarker	Salivary Levels in Patients	Findings
Cortisol	Increased levels [31,32]	Association between high levels of cortisol in early stages of the pathology [31,32] that correspond to peaks in pain, stress [33,34,35], and depression [36].
	Decreased levels [37,38]	Low cortisol levels are associated with the duration of the disease, and may be the cause of chronic adaptation to stress in fibromyalgia patients [37,39,40].
α-Amylase	Increased levels [33,41,42,43]	Fluctuations in amylase levels were observed depending on the timing of the sampling [42,44]. These changes may have been related to pain and stress in patients with fibromyalgia [33,45,46].
Transaldolasa	Increased levels [47,48]	Overexpression of transaldolase in FM-affected women that may have been due to decreased oxidative tissue damage [47]. Increased expression of transaldolase by two-dimensional electrophoresis was not useful to differentiate between FM and migraine [48].
	Unaltered levels [49]	Absence of changes in transaldolase expression in response to thermal treatments [49].
Phosphoglycerate mutase 1	Increased levels [47,48]	Overexpression of PGM1 determined by ELISA, Western blot, and total optical density [47]. This increase in PGM1 expression can also be detected in patients with migraine [48].
	Decreased levels [49]	FGM1 was significantly reduced in FM subjects after receiving mud-balneotherapy [49].
Calgranulina	Increased levels [47,50]	Increased levels of calgranulin A and C in fibromyalgia patients [47], which may be explained by their protective role against oxidative stress [50].

**Table 2:** Potential salivary biomarkers in fibromyalgia.

Biomarker	Fluid	Biomarker Levels in Fibromyalgia Patients	Findings
CRP	Serum	Increased levels [88,89]	CRP has been found to provide information on the inflammatory status of patients with FM [88,89,90].
IL-1β	Serum	Increased levels [87,91,92]	Higher serum levels were found in patients with fibromyalgia. No differences after adjustment of age as a covariate [91]. IL-1β, released by thalamic mast cells, contributes to inflammation and pain and can also stimulate thalamic nociceptive neurons [87].
		Unaltered levels [93,94]	No differences in serum levels between healthy and fibromyalgia patients [93,94].
	Skin biopsies	Increased levels [95]	There was presence of IL-1β in skin biopsies of fibromyalgia patients compared to healthy controls, which may be associated with an inflammatory component in the induction of pain [95].
	Muscle samples	Unaltered levels [96]	No differences between cytokine concentrations in healthy control compared to patients with fibromyalgia [96].
	Cerebrospinal fluid	Unaltered levels [93]	No differences of IL-1β were found [93].
IL-6	Serum	Increased levels [5,87,91,97,98,99,100,101]	Higher serum levels were found in patients with fibromyalgia, which was significantly associated with fatigue and pain ratings [5,100,101]. No differences after adjustment of age as a covariate [91]. Higher levels of IL-6 in peripheral blood of fibromyalgia patients were related to hyperalgesia [92]. IL-6 released by thalamic mast cells contributed to inflammation and pain and can also stimulate thalamic nociceptive neurons [87]. This elevation also contributes to the symptoms of fibromyalgia [98,99].
	Skin biopsies	Increased levels [95]	There was presence of IL-6 in skin biopsies of fibromyalgia patients compared to healthy controls, which may be associated with an inflammatory component in the induction of pain [95].
	Muscle samples	Unaltered levels [96]	No differences between cytokine concentrations in healthy control compared to patients with fibromyalgia [96].
IL-8	Serum	Increased levels [5,91,92,102,103,104]	Higher serum levels were found in patients with fibromyalgia. No differences after adjustment of age as a covariate [91]. Fatigue and depression were associated with high levels of IL-8 with the promotion of sympathetic pain [5,92,104]. This increase also suggests the presence of anti-inflammatory response and a link with clinical symptoms [102]. Wang el al. did not correlate this cytokine in relation to pain intensity [103].
		Unaltered levels [94]	Severity of pain was positively correlated with IL-8 [94].
	Cerebrospinal fluid	Increased levels [93]	Higher levels of IL-8 in serum and cerebrospinal fluid of fibromyalgia patients [93].
	Muscle samples	Unaltered levels [96]	No differences between cytokine concentrations in healthy control compared to patients with fibromyalgia [96].
IL-10	Serum	Increased levels [91,92,100,102,105]	Higher serum levels were found in patients with fibromyalgia. No differences after adjustment of age as a covariate [91]. This market was also positively correlated with various pain scores [100]. This increase also suggests the presence of anti-inflammatory response and a link with clinical symptoms [102].
IL-17	Serum	Increased levels [106]	Fibromyalgia patients showed elevated levels of IL-17a as well as positive correlations with levels of IL-2, IL-4, IL-10, TNF, and IFN-γ [106].
	Intracellular levels of mononuclear blood cells	Increased levels [99]	Higher intracellular levels of IL-17 in peripheral blood mononuclear cells in fibromyalgia patients [99].
TNF- α	Serum	Increased levels [87,91,92,98,99,102,105]	Higher serum levels were found in patients with fibromyalgia. No differences after adjustment of age as a covariate [91]. TNF-α released by thalamic mast cells contributes to inflammation and pain and can also stimulate thalamic nociceptive neurons [87]. This elevation also contributes to the symptoms of fibromyalgia [98]. This increase also suggests the presence of anti-inflammatory response and a link with clinical symptoms [99,102].
		Unaltered levels [94]	Severity of pain was positively correlated to TNF-α [94].
	Skin biopsies	Increased levels [95]	There was presence of TNF-α in skin biopsies of fibromyalgia patients compared to healthy controls, which may be associated with an inflammatory component in the induction of pain [95].
	Muscle samples	Unaltered levels [96]	No differences between cytokine concentrations in healthy control compared to patients with fibromyalgia [96].
Eoxatin-2	Serum	Increased levels [107]	Significantly increased circulating levels of eotaxin-2 in serum of fibromyalgia patients compared with healthy controls [107].

CRP: C-reactive protein; IL-1β: interleukin 1-β; IL-2: interleukin 2; IL-4: interleukin 4; IL-6: interleukin 6; IL-8: interleukin 8; IL-10: interleukin 10; IL-17: interleukin 17; IFN-γ: interferon-γ; TNF-α: tumor necrosis factor α.
